# Underweight is associated with mortality in patients with severe sepsis and septic shock

**DOI:** 10.1186/2197-425X-3-S1-A876

**Published:** 2015-10-01

**Authors:** SM Lee, JW Kang, YH Jo, K Kim, JH Lee, J Lee, KP Rim

**Affiliations:** Emergency Medicine, Seoul National University Bundang Hospital, Seongnam, Korea Republic of Korea; Emergency Medicine, St. Carollo General Hospital, Suncheon, Korea Republic of Korea

## Introduction

It is well known that obesity is associate with improved mortality in severe sepsis in western countries. However, the prognosis of the patients with sepsis in Asian countries might be different from that of western countries.

## Objectives

This study was performed to investigate the association of the body mass index (BMI) with the mortality in patients with severe sepsis and septic shock in an Asian country.

## Methods

A retrospective analysis of prospectively collected database of patients with severe sepsis and septic shock was performed. We classified the patients into four groups according to the basic criteria of the World Health Organization (WHO) guidelines as follows, underweight, < 18.5; normal, 18.5-24.99; overweight, 25-29.99; obesity, ≥ 30 kg/m^2^, respectively. The multivariate logistic regression was performed to investigate the association of BMI with 28-day mortality adjusting for the demographic data, comorbidities, laboratory results and Acute Physiologic and Chronic Health Evaluation II score.

## Results

A total of 1022 patients were included and the overall mortality was 20.6%. The mortality was 30.6% in underweight, 18.5% in normal, 17.1% in overweight, and 14.3% in obesity, respectively (p < 0.05). In the multivariate analysis, underweight is independently associated with mortality compared with normal BMI (odds ratio (OR), 1.63; 95% CI, 1.12-2.37). However, overweight and obesity are not associate with mortality (OR, 0.913; 95% CI 0.63-1.51 and OR, 0.59; 95% CI, 0.24-2.25, respectively).

## Conclusions

Underweight is associated with 28-day mortality and obesity is not associated with the prognosis in patients with severe sepsis and septic shock in an Asian country.

## References

[1] WHO (1995). Physical status: the use and interpretation of anthropometry. Report of a WHO Expert Committee. WHO Technical Report Series 854.

